# Densely attention mechanism based network for COVID-19 detection in chest X-rays

**DOI:** 10.1038/s41598-022-27266-9

**Published:** 2023-01-06

**Authors:** Zahid Ullah, Muhammad Usman, Siddique Latif, Jeonghwan Gwak

**Affiliations:** 1 Department of Software, Korea National University of Transportation, Chungju, 27469 South Korea; 2 Department of Computer Science and Engineering, Seoul National University, Seoul, 08826 South Korea; 3 Faculty of Health and Computing, University of Southern Queensland, Toowoomba, QLD 4300 Australia; 4 Department of Biomedical Engineering, Korea National University of Transportation, Chungju, 27469 South Korea; 5 Department of AI Robotics Engineering, Korea National University of Transportation, Chungju, 27469 South Korea; 6 Department of IT. Energy Convergence (BK21 FOUR), Korea National University of Transportation, Chungju, 27469 South Korea

**Keywords:** Computer science, Medical imaging

## Abstract

Automatic COVID-19 detection using chest X-ray (CXR) can play a vital part in large-scale screening and epidemic control. However, the radiographic features of CXR have different composite appearances, for instance, diffuse reticular-nodular opacities and widespread ground-glass opacities. This makes the automatic recognition of COVID-19 using CXR imaging a challenging task. To overcome this issue, we propose a densely attention mechanism-based network (DAM-Net) for COVID-19 detection in CXR. DAM-Net adaptively extracts spatial features of COVID-19 from the infected regions with various appearances and scales. Our proposed DAM-Net is composed of dense layers, channel attention layers, adaptive downsampling layer, and label smoothing regularization loss function. Dense layers extract the spatial features and the channel attention approach adaptively builds up the weights of major feature channels and suppresses the redundant feature representations. We use the cross-entropy loss function based on label smoothing to limit the effect of interclass similarity upon feature representations. The network is trained and tested on the largest publicly available dataset, i.e., COVIDx, consisting of 17,342 CXRs. Experimental results demonstrate that the proposed approach obtains state-of-the-art results for COVID-19 classification with an accuracy of 97.22%, a sensitivity of 96.87%, a specificity of 99.12%, and a precision of 95.54%.

## Introduction

In the last few years, the world has been witnessing the progressive contamination of COVID-19 pandemic around the world. Yet the trends are unclear, however, some researchers believe that this disease may persevere till 2024^1^. The efficient way to avert the COVID-19 outbreak within society is the accurate screening for early diagnosis of this disease. In general, the COVID-19 diagnosis can be carried out by considering one of the three tests. (1) RT-PCR test: the Reverse Transcription Polymerase Chain Reaction captures the viral RNA from nasopharyngeal swab or sputum^2^. The result of this test consumes almost twelve hours, which is not beneficial because COVID-19 positive patients ought to be recognised as early as possible. While the test arrangement needs some specific equipment and material, that are not accessible easily. At different points, the results of RT-PCR from several tests of the same COVID-19 patients were inconsistent and generated a high false-negative rate^3,4^. (2) Computed tomography (CT)-Scan: the assessment based on Computed Tomography is comprised of evaluating radiographic images from various angles. In most hospitals, the required equipment for assessment is not easily available as well as it consumes 15–20 minutes for a patient further to require CT decontamination time^5^. In addition, CT-scan-based mass assessment of COVID-19 is not suitable due to its radiation exposure and cost^6^. (3) Chest X-ray (CXR) based assessment: it involves the evaluation of radiographic images and inspection for diffuse reticular-nodular opacities and consolidation, with peripheral, and bilateral predominance^7^. For this type of assessment, the required equipment is less inconvenient and can be lightweight and transportable. The resources of CXR are more easily accessible as compared to CT-scan and RT-PCR tests. Moreover, the CXR based test consumes around 15-20 seconds for each patient^2^, which illustrates that CXR based assessment is one of the most cost/time effective tools. Therefore, in the diagnostic workup of patients, CXR is an extensively utilized imaging modality, due to its low cost, low radiation and its fast imaging speed^8^.

**Figure 1:**
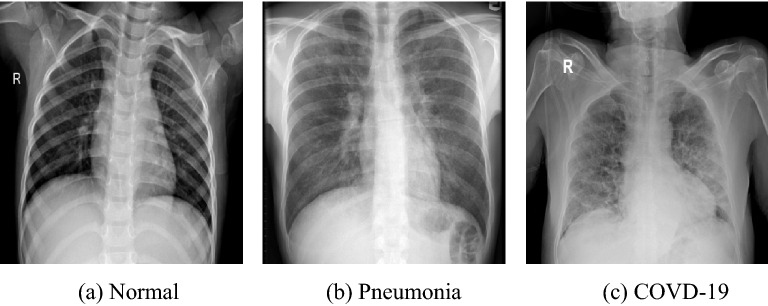
CXR for healthy and ill people from COVIDx datasets^9^, which classifies CXRs into classes of: (**a**) Normal case, (**b**) Pneumonia case, and (**c**) COVID-19 case.

Among the aforementioned techniques, RT-PCR^10^ is mostly utilized test because of its high specificity and sensitivity. Unfortunately, it is resource-intensive, laborious, expensive, and time-consuming^11^. Also, an RT-PCR test necessitates the use of skilled professionals who have been informed on how to use the RT-PCR kit to conduct this test, specifically, the use of nose or throat swabs for SARS-CoV-2 detection. Moreover, a complete setup is required for RT-PCR tests including laboratory, skilled practitioners, and RT-PCR machine for detection and inspection. In contrast, CXR offers a cost-effective, faster, more readily available, and time-saving diagnosis method^12^.

CXR imaging technique is mostly preferred over CT-scan, because the CT-scan imaging technique has high-dose radiation especially for pregnant women or children^13^. CXR on the other hand is having low radiation that shrinks the cross-infection risk and is available more widely than CT-scan. However, there are several drawbacks to the manual diagnosis of COVID-19 with CXRs. For instance, it is time taking and might be prone to human errors. Similarly, too many radiologists are needed for the manual diagnosis of COVID-19 in this pandemic situation. Therefore, an automated methodology is required to accurately diagnose COVID-19, and recently, numerous deep learning (DL) based approaches have been presented to achieve this goal^14^.

The recently proposed DL based techniques for COVID-19 diagnosis^9,15–17^ are categorized into two classes: segmentation-based methods and classification-based methods. The classification based approaches mainly extract discriminative features to classify the nature of pneumonia from raw CXRs^18,19^. Most researchers utilise convolutional neural networks (CNNs) architectures such as DenseNet^20^, ResNet^21^, and visual geometry group network (VGGNet)^22^ to learn feature representation from CXRs to accurately identify COVID-19^19^. However, the CNN geometric structures and the locations of sampling cannot be modified according to the composite shape of lesions^23^. From complex lesions, the robust learning of radiographic features is required for COVID-19 identification. The segmentation-based approaches examine the affected COVID-19 areas in the bilateral lung by training the network to detect the lung area and then the segmented image is fed to the classification network^17,24^. For instance, Oh et al.^17^ proposed a 2-phase model for COVID-19 detection using CXRs, in which the semantic segmentation has been carried out using DenseNet103 to detect lung contours, and the COVID-19 cases are classified using the ResNet-18 network. However, the segmentation-based approaches still have some limitations, such as the segmentation method being highly sensitive to complex shapes of the affected areas. Also, the performance mainly depends on the training data with an accurate annotation which is a time consuming task.

In COVID-19 patients, typical CXRs contain lung consolidation, ground-glass opacities (GGOs), and peripheral lung involvement, which have different irregular shapes (i.e., patchy, reticular nodular, diffuse, and hazy)^25^, as depicted in Fig. 1. Moreover, the lesion region size and position are highly varied at various steps of the infection and among different COVID-19 patients. This makes the development of the COVID-19 detection a more challenging task. Therefore, it is important to enabling the network to adaptively learn the affected areas with composite structures.

To address the aforementioned issues, in this paper, we present a densely attention mechanism-based network (DAM-Net) that can automatically learn important features by paying attention to the region of interest, such that reticular opacities, lung consolidation, and GGOs, and capture radiographic features robustly. Inspired by recent studies^26,27^ in computer vision, we enable the proposed DAM-Net to extract multiscale and key features to accurately identify the COVID-19. In our proposed DAM-Net, densely connected CNN blocks can capture and connect image characteristics at numerous scales in the spatial domain to capture high-level features. The channel attention mechanism in the channel domain is combined with DenseNet to pay attention to crucial parts. In addition, we use cross-entropy loss function based on label smoothing to effectively diminish the interclass similarity effect in classifying COVID-19.

## Related work

Many researchers have proposed DL  models for automatic COVID-19 diagnosis using CXRs. The obtained results are encouraging, however, there is still room to improve the performance. This section analyzes the related work based on deep learning where we analyzed the pros and cons of previous proposed models and discussed the attention mechanism.

### COVID-19 classification based on deep learning

The COVID-19 pandemic significantly increases the workload on doctors and other medical workers. Thus, to ease the burden on radiologists and to improve efficiency, researchers gradually adapt the recent developments of DL to interpret CXR images. For instance, Tabik et al.^28^ presented a three stages framework to categorize the CXRs into COVID-19 and non-COVID-19. Firstly, they employed a bounding box segmentation method to crop the significant lung region from the CXR images. Secondly, two-class inherent transformations are generated using a GAN-inspired class inherent transformation network (i.e., $x-$ and $x+$) from each input image *x*. Finally, they utilized a Resnet-50 for classification. They developed an aggregation strategy in their proposed framework to achieve the final output. However, as the number of classes increases, the number of generators will also increase, which need to be trained in the second phase of this method. Hence, the scaling of multi-class classification becomes difficult.

Shi et al.^29^ presented attention-based CNN model where they incorporated a framework of teacher-student transfer learning for COVID-19 detection by utilizing CXR and CT-scan. They collected 450 COVID-19 CXR from two different databases including COVID-19 X-ray dataset^30^ and the Italian Society of Medical and Interventional Radiology COVID-19 database^31^. Results are presented in recall (86.49%), precision (90.14%), F1-score (88.28%), and accuracy (87.98%) to assess the performance of the model. Wang et al.^9^ proposed COVID-Net to classify COVID-19, pneumonia bacterial, pneumonia viral, and normal CXR images. They have also introduced an open-access benchmark data called COVIDx dataset by combining five different publicly available data repositories^30,32–35^. They reported 83.5% overall accuracy (i.e., for four classes) and 92.5% accuracy for a 3-class classification tasks. They claimed 91.0% sensitivity rate for COVID-19. Islam et al.^36^ detected the COVID-19 disease in the CXR image using long short-term memory (LSTM) network. Initially, they extracted the deep features simply using CNN, then LSTM network is employed to classify COVID-19. They have considered different publicly available datasets that comprised of 4,575 total CXR and achieved a high-performance rate. However, the model is unable to differentiate the other CXRs views as it mainly focused on the posterior-anterior CXR view.

Degerli et al.^37^ proposed a model namely reliable COVID-19 detection network (ReCovNet) to detect COVID-19 out of 14 different thoracic disease using CXRs. The ReCovNet is evaluated using QaTa-COVID-19 dataset and obtained efficient results. Importantly, they compile the QaTa-COVID-19 dataset which was established in their previous study^38^. Likewise, Haghanifar et al.^39^ proposed a transfer learning approach called COVID-CXNet mainly to detect coronavirus-related features efficiently. They also illustrated the significance of Grad-CAM heatmaps by comparing model visualization over a batch sample and accuracy rate. The authors claimed that the COVID-CXNet obtain overall 87.88% accuracy.

Ozturk et al.^40^ presented a model called DarkCovidNet for multi-class classification and binary class classification. They achieved 87.02% accuracy for multi-class and 98.08% accuracy for binary class. Khan et al.^41^ proposed a DL model named CoroNet that automatically classifies COVID-19 disease from CXRs. They collected the CXRs from various publicly available sources which contain 310 normal images, 327 pneumonia viral images, 330 pneumonia bacterial, and 284 COVID-19 images. They achieve the accuracy of 89.5% using the proposed CoroNet. Mesut et al.^42^ developed MobileNet for COVID-19 detection. They have evaluated their model using CXR images and considered three different classes in CXR images, namely, pneumonia, COVID-19, and Normal. They preprocessed the whole dataset to reduce the noise using the fuzzy color technique and achieved 99.27% accuracy. However, the major drawback of this model is that it is unable to work effectively on low-resolution CXRs.

Most of the above discussed paper use CNNs to extract feature representations from CXRs to perform COVID-19 detection. The extraction of feature from the infected areas with composite shapes cannot be suitable enough because of the fixed geometric structures of CNN, the locations of sampling are fixed and cannot be changed according to the complex lesion shapes^23^. Hence, in COVID-19 infected patients, it is important to learn robust radiographic features from composite lesions. To overcome this issue, we have developed a DAM-Net that establishes rich context information of local features to pay attention to relevant infected areas, which immensely assists the network to learn radiographic features from complex lesions.

### Feature extraction and attention mechanism

In image processing, the feature extraction task is essential to reduce the redundant input information. Specifically, for COVID-19 detection, the extraction of efficient features is highly crucial. The algorithm may overfit and poorly generalize to new samples due to the poor features^43^. The conventional methods are not able to extract robust features from the complex data like COVID-19 CXRs. Recently, automatic feature extraction using deep neural networks are becoming very popular. For instance, the author in^44^, proposed a deep autoencoder to predict the COVID-19 patient’s survival probability. Moreover, the attention mechanisms^45^ are currently the essential building block of most state-of-the-art architectures, that accommodate more complex datasets and more flexible modeling representation. To help the model focus on important representation for CXR classification, we exploit the idea from the attention mechanism. The basic idea of the attention mechanism is similar to human perception. For instance, in human perception, attention plays a vital role, enabling humans to pay attention to an essential portion of the picture, rather than proceeding with the complete image in its entirety^46^. Due to its significance, researchers proposed attention approaches in the DL  field to enhance CNN’s performance in image segmentation and image classification tasks. For instance, Jie et al.^47^ developed a solid squeeze and excitation module to exploit the relationships between channels. They employed global average pooling to pool features and attain channel descriptors. Further, they used two fully connected layers to detect the relationships between channels.

The attention approach can be classified into spatial-wise and channel-wise attention modules. In channel-wise approach^47^, the attention module utilized an inter-channel relationship with extra convolutional layers, which denotes the correlation between the key information and the current channel. With the larger weights, we can pay more attention to the channel. In paper^47^, the authors proposed Squeeze-and-Excitation (SE) networks to illustrate the importance of each channel through various learned weights. On the other hand, in spatial-wise mechanism^46,48,49^, the attention module detects prominent features by using the inter-spatial relationship from various locations of feature maps. Max et al.^50^ presented a spatial transformer network to convert the feature map within the network spatially. Moreover, some researchers^51,52^, have concatenated both channel and attention approaches to take synergetic effects. The existing literature shows that DL models obtained good results in processing medical images. However, the current research rarely considers the role of the attention mechanism for COVID-19 classification. It is thus unable to capture the spatial-wise and channel-wise relationship in a variety of scopes.

Therefore, in this work, we employed SE^47^ based attention to choose prominent features adaptively by accommodating different feature weights in the channel domain. SE approach is an extensively studied approach with accessible software to add to any CNN for channel-wise weighting. In contrast to the previous studies^46,48–50^ that were focused mainly on the spatial attention having numerous weight parameters, this work employs the attention mechanism that focuses on channel attention with fewer parameters for COVID-19 classification.

## Proposed method

**Figure 2:**
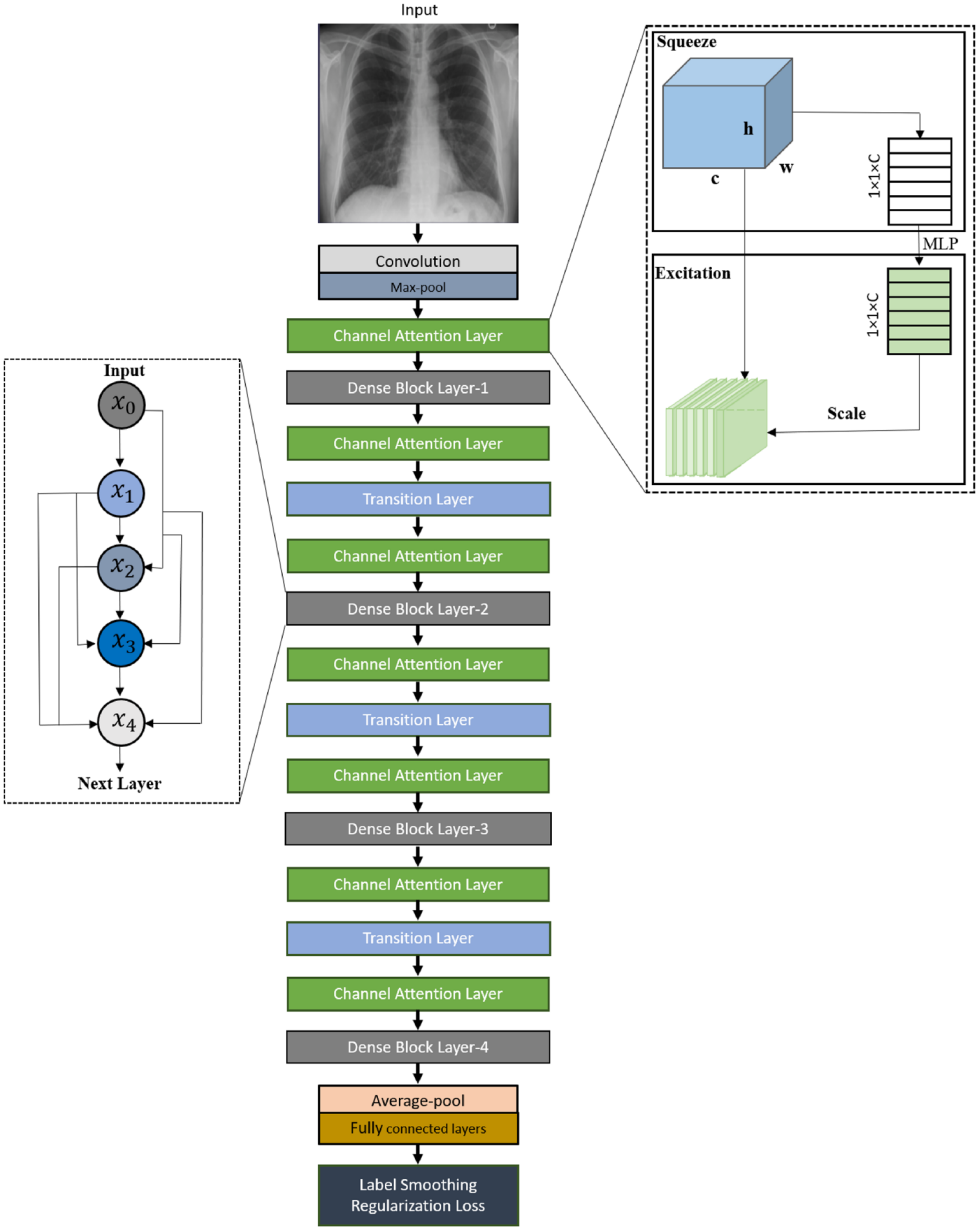
Our proposed COVID-19 classification model using CXRs, where the left hand side nodes $x_0$-$x_4$ represent densely connected convolution operation (Dense Block layer), while, the right hand side shows the channel attention layer.

In our proposed DAM-Net, we use Dense Block to extract spatial features at different scales. We exploit channel features attention-based squeeze-excitation block to adaptively selects prominent features by adjusting the weights of various feature maps in the channel domain. The cross-entropy loss is integrated with label smoothing to minimize the inter-class similarity effect. To specifically test the detection capability of the model in differentiating COVID-19 from other types of pneumonia and normal CXRs, we developed the 3-class (i.e., COVID-19 - for patients with COVID-19, normal - for healthy patients, and pneumonia - for patients with non-COVID-19 pneumonia) detection network. Fig. 2 shows the schematic diagram of the proposed DAM-Net.

### Spatial feature extraction

We employed DenseNet to capture spatial features of various scales. It is important to note that the features of various scales are further cross-linked by this densely connected structure, which achieves high performance than conventional CNNs in representing the complex semantic relationship of different diseases in CXRs (i.e., Normal, Pneumonia, and COVID-19^53^).

In contrast to the shallow network, DenseNet can learn discriminative and robust features to achieve better performance. It also solves the vanishing gradient problem by introducing feature reusability in the network. It enables learning spatial features by introducing direct connections from each layer to all subsequent layers. The densely block layer tries to maintain the smooth flow of information between the network layers^20^. Similarly, the ith layer $K_l$ receives the feature maps of all preceding layers as input and then proceeds its corresponding feature map to each succeeding layer:

1 $$\begin{aligned} {K_l} = M_{l,G} ([x_0,x_1,..., {x_l}_{-1}]), \end{aligned}$$

where, $M_{l,G}$ (.) illustrates a composite function comprising batch normalization^54^, ReLU^55^, pooling^56^, and convolution layer and $[x_0,x_1,...,x_{l-1}]$ represents the integrated feature map from layers $[0,...,l_{-1}]$. *G* is the growth rate that represents number of output feature maps. Cascading multiple layers of composite functions and feature map concatenations form a Dense Block (*L*, *G*), which has *L* layers and a growth rate of *G*. In Equation 1, the concatenation in the Dense Block causes the input size to be increased as the number of layers increases in Dense Block. After each Dense Block, a transition layer is used for downsampling. The transitional layer consists of a batch normalization layer of a $1 \times 1$ convolutional layer and a $2 \times 2$ average pooling layer. We extracted the complex spatial features using DenseNet121 which consists of 4-dense blocks with transition layer along downsampling, and it prevents the overfitting problem efficiently.

A densely connected pattern is employed in this structure, where it needs limited parameters as compared to traditional CNN. This network significantly reduces the requirement of learning unnecessary details, also diminishes the feature maps requires by the network layer. In this way, the efficiency of parameters is significantly enhanced. Whereas, the continuous concatenation of various layers needs every layer to approach the gradients from the input and loss function. The flow of information between layers gets improved due to this fast access and also the problem of gradient disappearance is reduced.

The main benefit of these tiny bonds among layers, adjacent to the input and output is to let the former features proceed backward efficiently for reconsideration of feature representations. Hence, it is possible to utilize this network structure to extract more meaningful features. The extracted features from all the layers can also be reprocessed to be fused to obtain a more informative descriptor, after which can be employed for various applications to achieve improved results^57^. This method connects numerous feature maps and has no intention for feature reconsideration in between every layer. As shown in Fig. 2, left-hand side, we managed the last layer as input for the next layer, rather than integrating all feature maps. Mostly, the structures of the traditional network are based on the connection of $L(L + 1)/2$, rather than the *L* connection. Based on the preceding layers, *lth* layer feature maps can be computed including $X_0,...,X_{l-1}$.

### Channel features’ attention-based on SE block

Channel attention was fundamentally introduced for classification problems having SE block^47^. In channel attention, the weights are trainable parameters and more specifically these weights are multiplied by each channel.

To improve the performance of our proposed DAM-Net, we utilized the attention-based SE blocks^47,58^ into the feature channel domain that adaptively chooses prominent features by adjusting the weights of various feature maps in the channel domain. The SE-based attention module can be utilized in pairs with any convolution layer to weight each channel to eliminate redundancy. This attention method captures the essential characteristics by rearranging the weights of various size feature maps in the channel realm^59^. In this study, we only combined the transition layers and the dense block in the channel attention approach, so that we can fully utilize the channel attention module without adding a high number of parameters. In addition, the channel attention network is a small and efficient architecture and more importantly, it will not cause an overfitting problem, because it adds only 0.21M parameters. The transition layer is comprises a convolution layer of $1 \times 1$ and an average pooling using stride 2 to minimize the feature maps accordingly. The adaptive downsample is the combination of the channel attention module and transition layer. The feature channel processing is classified into two phases (i.e., squeeze and excitation) as shown in Fig. 2 right-hand side.

### Squeeze module

In this phase, the input features are squeezed into a 1-Dimensional vector where the channels represent the length of this vector. In Eq. (2), $H \times W \times C$ shows the original size of input features *U*, while, $H \times W$ is the spatial domain size, and *C* represents the number of channels. Each spatial domain $H \times W$ is compressed to a value using global average pooling, hence, the input feature maps of size $H \times W \times C$ are reduced to a tensor of $1 \times 1 \times C$. The squeeze output $c_{th}$ element (i.e., $z_c$) is calculated as:

2 $$\begin{aligned} {z_c} = F_{sq}(u_c) = \frac{1}{W \times H} \sum _{i=1}^W \sum _{j=1}^H u_c(i,j). \end{aligned}$$

### Excitation module

In the excitation stage, the gate approach of two nonlinear fully connected layers is utilized to extract the dependencies between channels. The dimensions of these two layers are $\frac{C}{16}$ and *C*, to restrain the complexity of the model. The excitation module is a multi-layer perceptron (MLP) which consists of a single hidden layer. The output of excitation is represented as $s_c$ and computed as:

3 $$\begin{aligned} s_c = F_{ex}(z, W) = \sigma (g(z, W)) = \sigma (W_2\delta (W_1z)), \end{aligned}$$

where $\delta$ and $\sigma$ denotes ReLU and sigmoid functions, respectively, while, the parameters of *C* and $\frac{C}{16}$ layers are denoted by $W_1$ and $W_2$, respectively. Afterward, a corresponding weight is assigned to each feature channel.

4 $$\begin{aligned} u'_c(i,j) = s_c \times u_c(i,j), \end{aligned}$$

where the inputs $u_c$ and $s_c$ represent the original feature map and weight vector, respectively. The output $u'_c$ feature map is achieved via channel-wise multiplication. The channel attention module squeezes and expands the feature channels and allots adaptive weights to various features. This module significantly reduces the overfitting problem using limited parameters, which reduces the risk of overfitting, as compared to the attention model for feature maps. The SE-based attention does not affect the training time, due to its low computation burden. Fig. 2 right side illustrates the scheme of the placement of the SE blocks, as SE is a simple but powerful attention approach.

### Loss function

Finally, we combined the cross-entropy loss with label smoothing, to minimize the inter-class similarity effect. For COVID-19 classification, we have added a ReLU activation function to the final layer to calculate the probability. We compute the loss value by providing the maximum probability as an input to the cross-entropy function. Usually, in a one-hot vector, the class vector is transformed, where one element is 1 and the remaining elements are 0, for an n-length array. In this work, we employed label smoothing^60^ to enhance the loss function of the original cross-entropy. The cross-entropy predicted value between the network output $L_i$ and target $y_i$ is computed using backpropagation,

5 $$\begin{aligned} H(y,p) = \sum _{i=1}^I -y_i log (p_i), \end{aligned}$$

where in Eq. (5), the label of $y_i$ is 1 which is the true category, and 0 for the remaining category. Specifically, the loss with label smoothing just examines the correct label position loss. Ignoring the wrong labels position loss compels the model to give excessive surveillance to improve the likelihood of correct label prediction, rather than minimizing the likelihood of wrong label prediction. We used label smoothing in the training sample, to examine both the incorrect and correct label positions loss, such as:

6 $$\begin{aligned} y' = (1- \epsilon )y + \epsilon u(I), \end{aligned}$$

where in Eq. (6), $y'$ represents the obtained sample after the operation of label smoothing, regarding class *I*, the *u*(*I*) follow a uniform distribution, $\epsilon$ shows the smoothing factor. Hence, the cross-entropy loss provides attention to both the loss of correct class and other classes.

## Experimental setup

### Dataset details

We have utilized a publicly available COVIDx dataset (https://github.com/lindawangg/COVID-Net/blob/master/docs/COVIDx.md) that was initially comprised of 13,975 CXRs across 13,870 patient cases and has been updated time by time^9^. This dataset is a combination and modified form of five other open-access data repositories such as (a) COVID-19 radiography database^61^, (b) COVID-19 CXR Dataset^35^, (c) ActualMed COVID-19 CXR Dataset Initiative, established in collaboration with ActualMed^33^, (d) COVID-19 CXR Dataset Initiative^62^, and (e) COVID-19 Image Data Collection^63^.

**Figure 3:**
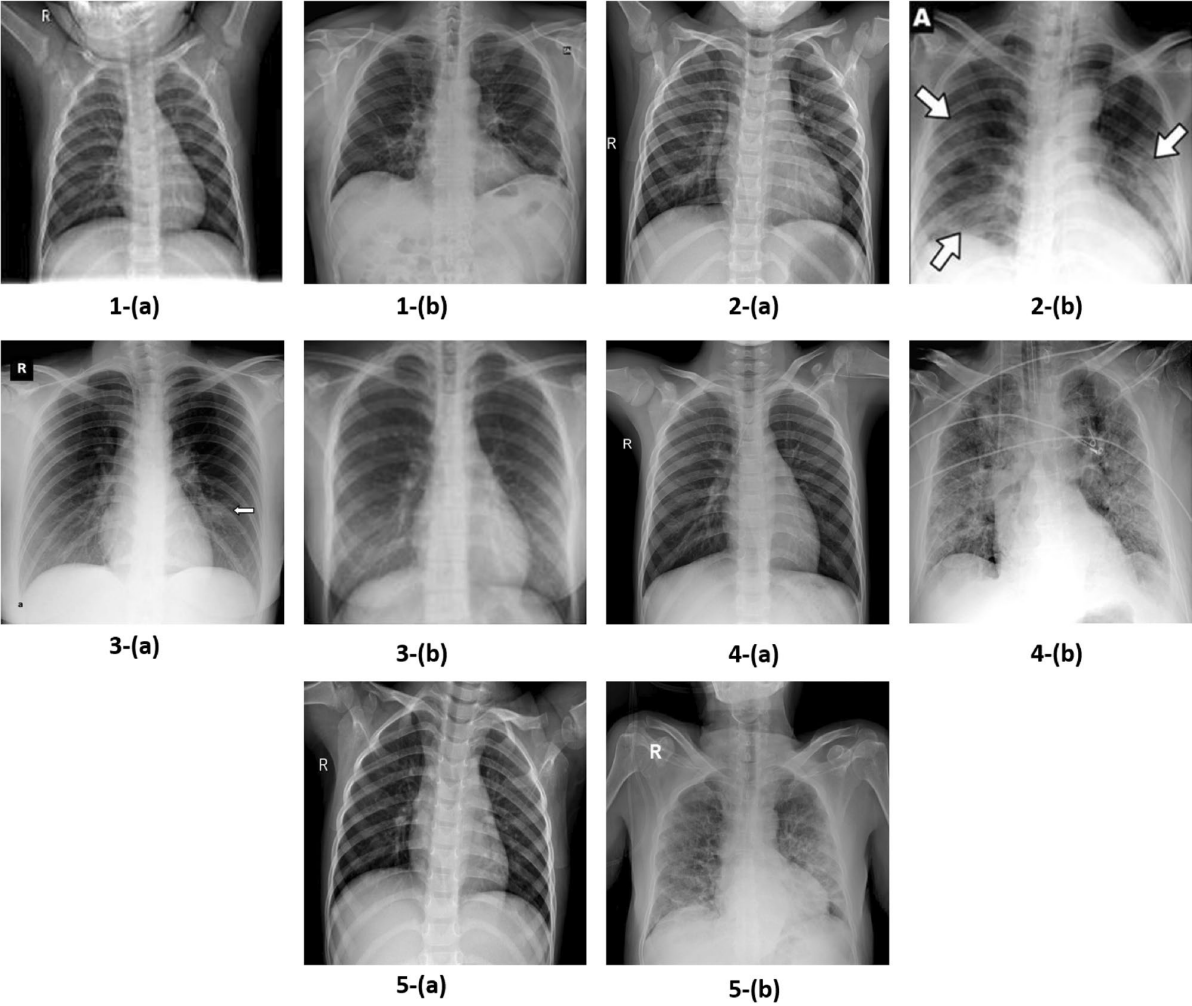
Sample images of COVIDx dataset from various repositories, where (**a**) represent normal image and (**b**) represent COVID-19 image from each repository, such as, 1. ActualMed COVID-19 CXR Dataset Initiative^33^, 2. COVID-19 radiography database^61^, 3. COVID-19 CXR Dataset Initiative^62^, 4. COVID-19 Image Data Collection^63^, and 5. COVID-19 CXR dataset^35^.

**Figure 4:**
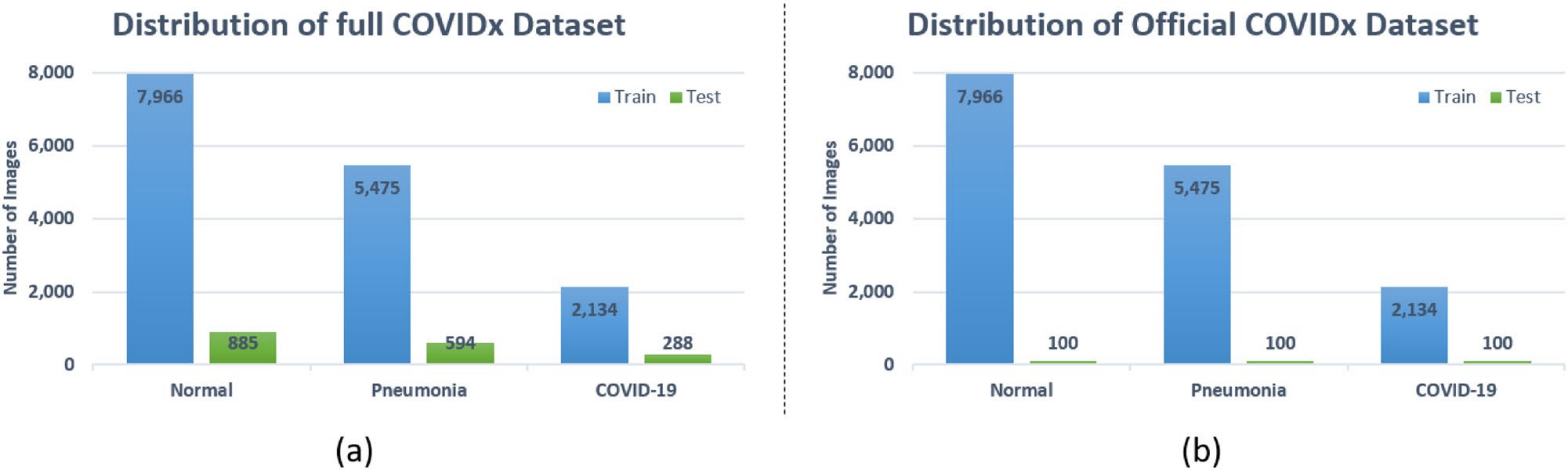
Class distribution of COVIDx dataset, where normal means no infection. Right bar depicts number of test CXRs, left bar depicts number of training CXRs.

The images of each dataset is illustrated in Fig. 3. There are three classes in COVIDx dataset: COVID-19 (X-Rays with positive COVID-19), Pneumonia (CXRs that consist of some form of viral or bacterial pneumonia, but no COVID-19), and Normal CXRs. Currently, there are two versions of the COVIDx dataset (i.e., 1. Official COVIDx and 2. Full COVIDx that differs only in the test set. During the creation of this dataset, some images of the COVID-19 radiography database^61^ were not available. Therefore, the dataset distribution varies from the official dataset as shown in Fig. 4. To further understand the creation of the official dataset, please refer to the link (https://github.com/lindawangg/COVID-Net/blob/master/docs/COVIDx.md). In this work, we have considered the full COVIDx dataset which is a huge open-access benchmark dataset concerning the number of positive COVID-19 cases, that contains 6,069 pneumonia cases, 8,851 normal cases, and 2,422 COVID-19 confirmed positive cases. Fig. 4a, shows the distribution of training and testing data of our experimental work. During experimentation, we follow the data usage agreement provided by COVIDx dataset (https://github.com/lindawangg/COVID-Net/blob/master/LICENSE.md) and all the experiments were carried out in accordance with relevant guidelines and regulations.

### Data pre-processing

The COVIDx dataset contains 2D CXRs in the jpg and png formats. Also, the resolution and contrast of the images in the COVIDx dataset are not the same, because the dataset is made up of images from several sources. Therefore, we used contrast limited adaptive histogram equalization^64^ to alleviate the noise due to contrast distribution. We scaled the CXRs to fit the input resolution ($224 \times 224$) of the proposed network. Initially, each image was uniformly scaled to the smallest original dimension, afterward, a central crop was applied to preserve the aspect ratio of the original images. Moreover, standard augmentation such that rotation ($\pm 10 \%$), translation ($\pm 10\%$), and zoom ($\pm 15\%$) have been applied to reduce the imbalance in the dataset.

### Training strategy

The proposed DAM-Net has been implemented in the Pytorch framework and traning is performed on NVIDIA RTX-3090 GPU and Intel Core-i5-12400F CPU 3.7 GHz with 16 GB RAM. The hyperparameters and settings of our network are as follows: we resized all radiographs to $224 \times 224 \times 3$ as the input and set the batch size as 12. After each epoch, the accuracy was calculated for validation. If after fifty epochs, the improvement in terms of validation accuracy was not significant, the learning rate was reduced to half and the model was replaced with the best of fifty epochs. We utilized the Adam optimizer and the minimum value for the learning rate was set to 0.001. During the training process, to get a stable distribution of activation values, each convolutional layer is supported by a batch normalization layer^54^. Mainly, the non-linearity layer (i.e., ReLU) is used after the batch normalization layer. It is noteworthy that we use the ReLU activation over a hyperbolic tangent and leaky ReLU due to its better performance on the validation set. Additionally, the early stopping strategy is employed^65^ to avoid over-fitting. This technique halts the training process once it detects not any change in the validation loss value, thus reducing the chances of overfitting of the network on the training data.

### Evaluation metrics

We utilized various evaluation parameters to evaluate the performance of our proposed method for COVID-19 classification such as Accuracy, Sensitivity, Specificity, and Precision.

#### Accuracy

Accuracy calculates the proportion of images that are identified correctly.

7 $$\begin{aligned} Accuracy = \frac{TP + TN}{TP + TN + FP + FN} \end{aligned}$$

#### Sensitivity

Sensitivity is the ratio of the positive cases that have been correctly detected to all the positive cases.

8 $$\begin{aligned} Sensitivity = \frac{TP}{TP + FN} \end{aligned}$$

#### Specificity

Specificity is the ratio of the negative cases that have been correctly classified to all the negative cases.

9 $$\begin{aligned} Specificity = \frac{TN}{TN + FP} \end{aligned}$$

#### Precision

This metric quantifies the number of correct positive predictions.

10 $$\begin{aligned} Precision = \frac{TP}{TP + FP} \end{aligned}$$

TP, TN, FP, and FN illustrate the total number of true positive, true negative, false positive and false negative, respectively.

## Results and discussion

In this Section, we present the results on COVID-19 detection using DAM-Net. We performed training and evaluation using the publicly available COVIDx dataset^9^. Results from multiple experiments are explained below.

### COVID-19 detection

**Figure 5:**
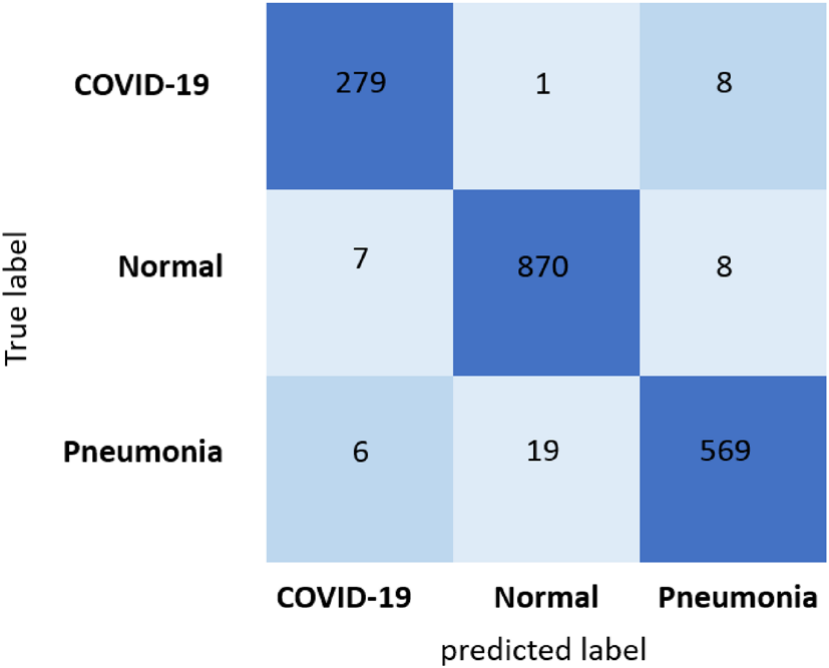
Confusion matrix of our proposed model: The dark-blue boxes illustrates the true prediction, and the light-blue boxes illustrates the false prediction.

We evaluated the proposed DAM-Net for COVID-19 detection and results are compared with recently DL-based methods^17–19,40,66–69^. For COVID-19 prediction, the DAM-Net is trained on 2,134 COVID-19 infected CXRs, while 288 COVID-19 cases are considered for testing from 13,870 patients as illustrated in Fig. 4, and achieve 97.22%, 96.87%, 99.12, and 95.54% accuracy, sensitivity, specificity, and precision rate, respectively. In contrast to the previous methods, our proposed DAM-Net achieve considerably better performance as highlighted in Table 1. The confusion matrix for the proposed DAM-Net is illustrated in Fig. 5, which shows that only nine out of 288 CXRs of COVID-19 are not screened out, and thirteen out of 1,767 CXRs are mistakenly considered as COVID-19. This shows that the ratio of error is minor in contrast to the total number of CXRs for DAM-Net.

### Comparison with state-of-the-art methods

To show the effectiveness of proposed DAM-Net, we compare the results with previous state-of-the-art COVID-19 detection techniques in Table 1. Following studies are selected for comparison. COVID-Net^9^: a tailored deep convolutional neural network that utilizes a pattern of projection-expansion-projection design. EDL-COVID^67^: is a snapshot ensemble deep learning model based on COVID-Net^9^ that consists of snapshot model training and model ensembling. Deep-COVID^18^: a transfer learning-based technique is used, which exploits the pre-trained networks such as, ResNet18, ResNet50, SqueezeNet, and DenseNet-121 for COVID-19 detection. CoroNet^68^: a semi-supervised learning technique based on autoencoders is used, which perform task base feature extraction and COVID-19 identification. PbCNN^17^: a patch-based CNN with comparatively less number of trainable parameters is used for COVID-19 identification. DarkCovidNet^40^: a YOLO (you only look once) based classifier without using transfer learning strategies is utilised for COVID-19 CXRs classification.

nCOVnet^70^: a transfer learning-based deep neural network that comprised of 24 layers.

As depicted in Table 1, our proposed method surpasses the existing approaches by a huge margin on all metrics. In comparison to the second-best model (i.e., EDL-COVID^67^) in Table 1, the DAM-Net has significant performance improvements, with the accuracy improved by 2.22%, sensitivity improved by 0.87%, and the precision improved by 1.44% in the COVIDx^9^ dataset. To show the recognition result of DAM-Net in the COVIDx dataset, we utilized the confusion matrix as shown in Fig. 5, which further validates our model’s performance where we achieve higher true positives for all individual categories. The proposed model misclassified nine COVID-19 cases only in the COVIDx dataset. The classification results validate that for COVID-19 radiological images our method can efficiently extract discriminative features and make a comparatively high accurate prediction for automatic diagnosis. Experimental results shows that our proposed model is capable of handling various complex lesions of COVID-19 robustly by incorporating the advantages of the channel attention approach for learning major feature representations and suppressing the redundant features, which is superior to the existing methods.

**Table 1:** Comparison of our proposed method with existing state-of-the-art methods using COVIDx dataset, the best performances are indicated in bold.

Method	Accuracy (%)	Sensitivity (%)	Specificity (%)	Precision (%)
Zhong et al.^66^	86.94	76.38	95.83	78.01
Deep-COVID^18^	82.83	71.18	93.95	69.49
CoroNet^68^	89.02	90.62	95.36	79.09
EDL-COVID^67^	95.0	96.0	95.35	94.1
PbCNN^17^	91.9	92.5	96.4	76.9
Ismael et al.^19^	91.3	95.0	94.0	88.8
COVID-Net.^9^	93.3	91.0	99.4	98.9
Brunese et al.^69^	84.6	67.0	95.5	88.1
nCOVnet^70^	87.3	82.0	96.0	91.1
DarkCovidNet^40^	88.6	89.0	97.5	94.6
Rajaraman et al.^71^	82.6	64.0	96.0	88.8
**DAM-Net**	**97.22**	**96.87**	**99.12**	**95.54**

### Robustness analysis

To further prove the robustness of the proposed scheme, we also performed a cross-dataset validation. Here, we leverage the fact that COVIDx^9^ dataset consists of five different datasets: (a) COVID-19 radiography database^61^, (b) COVID-19 CXR Dataset^35^, (c) ActualMed COVID-19 CXR Dataset^33^, (d) COVID-19 CXR Dataset Initiative^62^, and (e) COVID-19 Image Data Collection^63^. For cross-dataset evaluations, we train our model on four datasets from COVIDx and use the remaining one for test purposes. We repeated this experiment three times while changing the test dataset to COVID-19 Image Data Collection^63^, COVID-19 CXR Dataset Initiative^62^, and ActualMed COVID-19 CXR Dataset^33^. The results for each set have been presented in Table 2. The results show that the proposed approach has remarkable generalization ability and able to detect COVID-19 in cross-data evaluations.

**Table 2:** Cross-dataset evaluation results for COVID-19 detection.

Dataset Repositories	Dataset Distribution		Accuracy (%)
	COVID-19	Non-COVID	
Covid-19 Image Data Collection^63^	187	73	93.07
Actualmed COVID-19 Chest X-ray Dataset Initiative^33^	58	127	94.59
COVID-19 Chest X-ray Dataset Initiative^62^	55	0	90.90

### Qualitative analysis using grad-CAM and attention map

**Figure 6:**
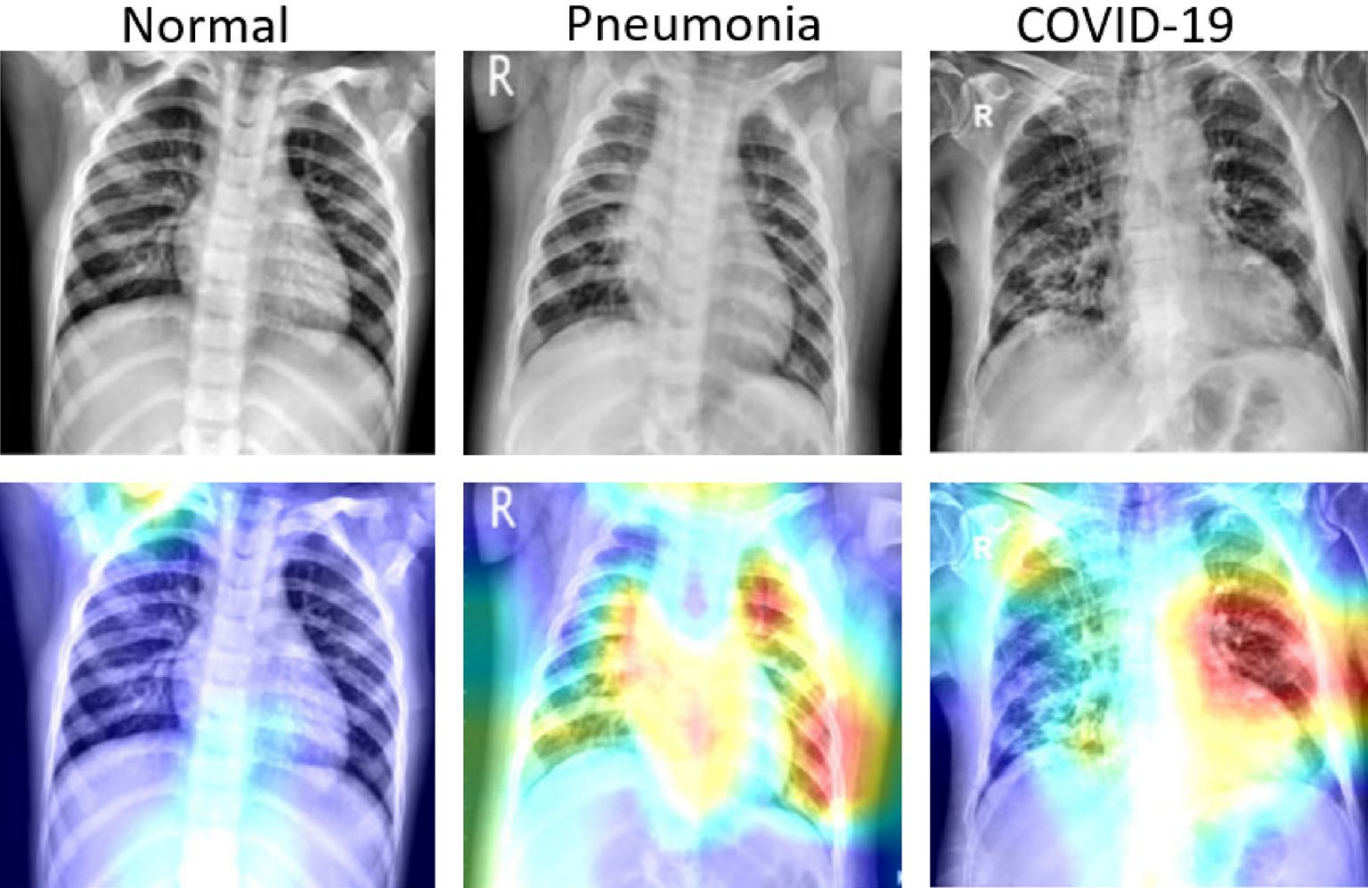
Gradient-based localization using Grad-CAM of Normal case, Pneumonia case, and COVID-19 case.

Although the quantitative effectiveness of our proposed model is evident from Table 1 in detecting COVID-19 from CXRs. It is also paramount to compare the classification results to clinical evidence in order to be useful in clinical practice. To this end, we utilized Gradient-weighted class activation mapping (Grad-CAM) to visualize normal, pneumonia, and COVID-19 cases as illustrated in Fig. 6. Grad-CAM is a renowned tool that is commonly employed to generate a localization map that highlights the prominent parts which help the network in predicting a class. From Fig. 6, the discriminative regions of interest can be seen localized in the normal, pneumonia, and COVID-19 cases.

**Figure 7:**
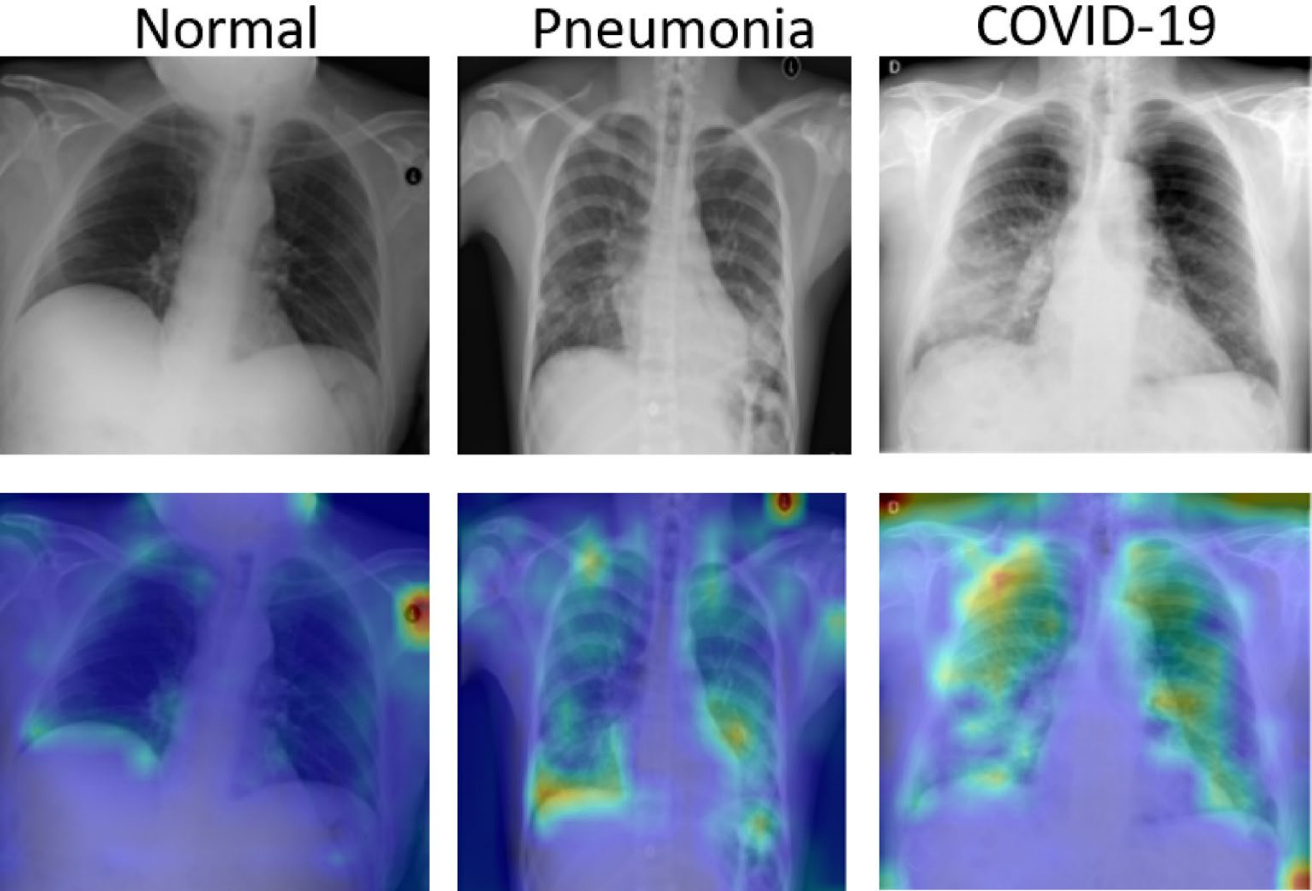
The first row: original images; second row: attention map obtained by our proposed DAM-Net.

On the other hand, our calculated attention maps successfully highlight more detailed infected parts as illustrated in Fig. 7 while other techniques fail to detect the prominent features. We can verify from Fig. 7 that our model is not making decisions based on inappropriate parts of CXRs. It can be seen that the focus areas for pneumonia and COVID-19 are evidently different from the normal case. We observe that our network pays more attention to different regions when classifying pneumonia and COVID-19. Moreover, exact conclusive feature detection is critical for both rapid confirmation and model interpretation of the reliability of outcomes. The attention map highlights the prominent parts of the CXRs and offers an explainable result of a prediction model. It provides insight to clinicians for accurate diagnosis and correcting the potential misdiagnosis in an AI-based model.

### Ablation studies

In this section, we have performed ablation studies on the COVIDx dataset to assess the effectiveness of every component. We mainly focused on the accuracy, sensitivity, specificity, and precision of the COVID-19 positive class in the COVIDx dataset. To analyze the proposed DAM-Net contributions, Table 3 quantitatively depicts the baseline model performance and our proposed approach. On the *1st*, *2nd*, and *3rd* row, results of the backbone DenseNet without both channel-attention and label smoothing, without Channel-Attention, and without label smoothing have been shown, respectively. We have presented the results of the proposed approach on the *4th* row of Table 3 to illustrate the composition method validity of these functional methods. Without both Channel-Attention and Label Smoothing, the network obtain the least results. Whereas, without channel attention only, the network generated competitive results. In contrast, with channel attention, the accuracy of the proposed DAM-Net is enhanced by 1.59%. The main reason for this enhancement is the channel attention that suppresses the representation of less useful features and enhances the representation of important image features.

**Table 3:** Ablation study metrics on full COVIDx dataset.

	Accuracy (%)	Sensitivity (%)	Specificity (%)	Precision (%)
Without Channel-Attention and Label Smoothing	92.26	90.35	93.24	90.35
Without Channel-Attention	94.05	89.23	98.17	90.49
Without Label Smoothing	95.64	92.01	98.91	94.30
DAM-Net	97.22	96.87	99.12	95.54

It is understood that every single module contributes to the promotion of performance. We can notice in Table 3, that the proposed DAM-Net outperforms the other ablation models. Hence, both the components bring enhancement and work efficiently with joint network structure. Due to our proposed network’s strong feature extraction capabilities, label smoothing also had a good influence on classification, which is increased by 1.58%. The label smoothing in other networks (i.e., VGGNet, ResNet, and ResNeXt) can be employed to achieve an improved classification accuracy.

## Conclusions and future works

In this work, a densely attention mechanism based network (DAM-Net) is presented for automatic detection of COVID-19 in CXRs that reached the state-of-art on the COVIDx dataset. In DAM-Net, we utilised DenseNet to capture spatial features of various scales and attention mechanism to focus on important attributes to accurately COVID-19 identification. Specifically, we showed that the DenseNet has strong feature representation and information extraction capabilities that help improve the performance. With the inclusion of a channel attention mechanism, the proposed model further improves the extraction of key features. The proposed model has been evaluated using the publicly available COVIDx data and demonstrated that our methodology outperforms the state-of-the-art models. We also evaluated the proposed model in cross-data setting, which show that DAM-Net has better generalization ability to perform cross-dataset COVID-19 identification. As future work, we intend to explore more attention approaches and will extend this model with sub-types of pneumonia, other lung diseases to learn definitive patterns that can assist radiologists.

## Data Availability

All data used in this paper is available online in the repository at https://github.com/lindawangg/COVID-Net/blob/master/docs/COVIDx.md.

## Code availability

All of the code necessary to reproduce our experimental findings can be found at https://github.com/AMIfirstLab/DAM-Net.
